# Caring for Children With Medical Complexity: A Clinical, Patient-Focused Curriculum

**DOI:** 10.15766/mep_2374-8265.11380

**Published:** 2024-01-30

**Authors:** Brittany Lattanza, Divya Lakhaney, Theresa Scott, Ashley Croker-Benn, Mirna Giordano, Sumeet L. Banker

**Affiliations:** 1 Fellow, Department of Pediatric Nephrology, Icahn School of Medicine at Mount Sinai; 2 Assistant Professor, Department of Pediatrics, Columbia University Irving Medical Center; 3 Assistant Professor, Department of Pediatrics, Weill Cornell Medical Center; 4 Second-Year Student, Mailman School of Public Health, Columbia University Irving Medical Center; 5 Associate Professor, Department of Pediatrics, Columbia University Irving Medical Center

**Keywords:** Complex Care, Clinical Teaching/Bedside Teaching, Curriculum Development, Pediatrics, Editor's Choice

## Abstract

**Introduction:**

Caring for children with medical complexity (CMC) requires specialized knowledge and skills. However, no standardized curricula are used across training programs as institutions have varying needs and resources.

**Methods:**

We created a patient-focused, interactive curriculum for two CMC topics: feeding/nutrition and pain/irritability. We integrated the 45-minute sessions into morning protected patient-care time on an inpatient pediatric team at an urban tertiary care hospital. Targeted toward all pediatric residents and medical students rotating in inpatient pediatrics over a 12-month period, the sessions used a mix of didactic, discussion, and hands-on activities. Learners on one of two inpatient teams received the curriculum, while those on the other received a curriculum unrelated to CMC and served as a control group. Both groups completed retrospective pre/post self-assessments to evaluate self-efficacy with respect to the learning objectives.

**Results:**

Over the 12-month period, 72 surveys were completed for the feeding/nutrition session, 78 surveys for the pain/irritability session, and 42 control surveys. The intervention group saw the greatest increase in self-efficacy scores generally in the feeding/nutrition session. All eight learning objectives saw significant improvement in self-efficacy scores for the intervention group. There was significantly greater improvement in self-efficacy for the intervention group compared to the control for all eight learning objectives.

**Discussion:**

Through this patient-focused curriculum, learners had improved self-efficacy scores compared to the natural learning occurring on the inpatient service. The curriculum could be adapted to fit the needs of other institutions and provides a practical, hands-on approach to learning about caring for CMC.

## Educational Objectives

By the end of this activity, learners will be able to:

1. Identify different types of enteric feeding tubes and describe the clinical scenarios in which they are used.
2. List the different types of formulas and describe the clinical scenarios in which they are used.
3. Discuss risks and benefits of feeding tube placement.
4. Identify steps to troubleshoot common problems associated with feeding tubes.
5. Outline components of the history and physical examination that aid in evaluating pain in children with medical complexity (CMC).
6. List different pharmacologic and nonpharmacologic interventions for pain in CMC.
7. Outline components of the history and physical examination that aid in recognizing and evaluating irritability in CMC.
8. List different pharmacologic and nonpharmacologic interventions for irritability in CMC.

## Introduction

Children with medical complexity (CMC) represent a growing patient population characterized by having chronic conditions, functional limitations (often associated with dependence on technology), high health care utilization, and substantial health care service needs.^1^ It is estimated that CMC account for less than 1% of the pediatric population but for over one-third of medical care for children, given their frequent hospitalizations and readmissions.^2,3^ With advances in medicine and technology, survival rates are increasing for infants born prematurely, those born with congenital anomalies, and/or other chronic and complex conditions. Given this growing patient population, it is essential for pediatric providers and trainees to know how to effectively care for this group of children. Despite this need, there is currently no standardized curriculum across residency programs to train pediatric residents in caring for CMC.^2^ Challenges that trainees have identified when it comes to caring for CMC include lack of care coordination, complex technology, psychosocial needs, and lack of effective training.^2^ Across academic primary care practices, provider confidence in caring for those with increasing needs was lower in resident–patient encounters as compared to attending–patient. Residents also reported fewer encounters with patients with complex needs than did attendings, due to a variety of reasons. Having fewer direct patient encounters may contribute to the lack of confidence that trainees have in caring for patients with complex needs. This suggests that residency programs need to create an educational model that focuses on specific patient encounters to directly address this lack of confidence in caring for CMC.^4^

A panel of North American CMC experts identified 11 essential topics in complex care for pediatric residents: feeding difficulties, pain/irritability, transition, feeding tube management, difficult discussions, team management/care coordination, dysmotility, aspiration, safety/emergency planning, neuromuscular/skeletal issues, and advocacy.^5^ Pediatric training programs have taken varied approaches to try to address this educational need, such as developing asynchronous learning modules on a variety of topics including, but not limited to, autonomic dysreflexia, pediatric spasticity, enteric feeding tubes, and ventriculoperitoneal shunts.^6–9^ Others have created an interactive teaching session on special considerations in performing a history and physical exam of CMC, including a video example of a physical exam.^10^ Conroy and colleagues created an asynchronous module on enteric feeding tubes featuring an in-depth self-study approach to learning enteric feeding tubes.^8^ As an elective for pediatric trainees, Kaushik developed a complex care curriculum that also included feeding in CMC as one of its topics.^11^ That curriculum was also anchored in experiential learning through patient care and site visits that served families of CMC; however, the didactic learning was asynchronous and unrelated to the patients the learners were serving.^11^

To address the need for formal teaching of caring for CMC, while recognizing that programs have different levels of faculty expertise and resources, we developed a patient-focused, interactive curriculum on specific topics in caring for CMC targeted to pediatric residents of all training years and medical students during their inpatient pediatrics rotation. Previous published curricula have relied on asynchronous learning modules or didactic sessions not necessarily anchored in the patients to whom the trainees were providing care. Thus, we sought to create a clinical curriculum grounded in the learners’ patients that could be easily integrated into the clinical space, while providing knowledge and skills that could be directly applied to their patients’ care.

Patient-centered learning allows learners to see patients in a more holistic and realistic manner, and therefore, help them to develop the skills to effectively partner with patients, families, and other members of the health care system.^12^ It has been proposed that patient-centered medical education should be defined as being “about the patients, with the patients, and for the patients, to ensure current and future doctors remain sensitive to all of the needs of the people for whom they care.”^13^ Students in a patient-centered curriculum have indicated that this form of learning allows them to appreciate the psychosocial aspects of disease that go beyond the clinical setting.^14^ Grounding our curriculum in the clinical experience allows learners to appreciate additional factors that go into the care of CMC, setting this curriculum apart from previous educational initiatives.

## Methods

### Development

The study team was guided by Kern's six-step model for curriculum development in the design of this curriculum.^15^ The team comprised pediatric hospital medicine faculty and one fellow, the director of our institution's complex care program, and a pediatric resident. A general needs assessment and thorough literature review highlighted both the nationwide need for more formalized teaching of how to care for CMC and the lack of existing standardized curricula. Eleven essential topics were identified as crucial to pediatric training.^5^ Our pediatric residency program had not previously had a formal curriculum on CMC. Learning targeted towards CMC occurred on rounds and was dependent on learners raising questions or faculty initiating a discussion on the topic. We distribued a needs assessment of targeted learners to pediatric residents of all training levels at a single residency program, asking them to rank the 11 topics from highest to lowest need. We met with key collaborators, including the residency program leadership and leaders of our pediatric complex care program, to identify gaps and opportunities in resident training. Feeding/nutrition and pain/irritability in CMC were identified as the top topics of interest and need for residents. Topics such as palliative care and outpatient care coordination were excluded as trainees received training on them in other settings. We developed goals and objectives based on the literature review, discussions with key collaborators, and feedback from the local needs assessment.

Several educational strategies, including readings, lectures, and role-plays, were considered.^15^ We ultimately settled on a multidisciplinary interactive and hands-on session that was anchored in real-time clinical experiences (i.e., a patient whom the learners were currently caring for on their inpatient service). This strategy was rooted in Kolb's experiential learning cycle, which uses the lived clinical experience as a starting point for reflective observation, receipt of additional knowledge and skills, and application of these in future clinical encounters with CMC.^16^

### Implementation

Prior to implementing the curriculum, the study team identified a group of content facilitators, including members of the study team as well as additional pediatric hospital medicine and gastroenterology faculty, a nurse practitioner from our local complex care program, and registered dieticians who had expertise in caring for CMC. This group met to discuss the curriculum vision, learning objectives, and session timing. We created a general facilitator's guide that provided an overview of the session (Appendix A). We also created resources that included a copy of the learning objectives, suggested prompts to help guide the session, and a sample case for each topic highlighting how to incorporate the prompts (Appendices B–E). A member of our curriculum implementation team scheduled the facilitators, identified a learning space, and helped identify the patient to be discussed for each session. The patient met criteria for medical complexity, was cared for by the learners on their inpatient team, and, if possible, was admitted to the hospital at the time of the session. Throughout the academic year, a member of the curriculum team reached out to potential facilitators and created a schedule of teaching availability. The team sent reminder emails approximately 1 week prior to the session. On the day before the session, a member of the curriculum team ensured a patient had been identified and sent an email reminder to all facilitators as well as to the inpatient team receiving the curriculum.

Sessions took place on our pediatric hospital medicine teaching service at an urban tertiary care center with a high case mix index and large volume of CMC. At our academic training institution, this service was made up of two inpatient pediatric teams where pediatric residents and medical students rotated. Patients on both teams were nearly identical with respect to the level of medical complexity; there was no separate complex care service. We implemented our curriculum for one of the two inpatient teams. The other inpatient team simultaneously received a curriculum unrelated to CMC, serving as a natural control group. We implemented the program starting in May 2021.

Residents spent 4 weeks on the inpatient rotation. Over the course of the 4 weeks, two CMC sessions occurred, one on feeding/nutrition (during week two of the rotation) and the other on pain/irritability (during week four). Each session was 45 minutes long. Morning clinical rounds started 1 hour earlier to accommodate the learning session, which required the attending faculty to come in 1 hour early on those days. Residents did not attend the program-wide morning conference on these days, to allow rounds to start earlier. The learning session occurred during the morning work time, immediately after hospitalist rounds had concluded. These accommodations in scheduling allowed the sessions to be integrated into this morning team rounding time and therefore anchor them in the clinical experience. The morning was a protected patient-care time at our institution, which ensured that residents did not miss the session due to their afternoon continuity clinic or other patient-care tasks arising over the course of the day.

Prior to the session, the facilitator received session materials, which included a general facilitator's guide (Appendix A). They also were sent materials pertinent to the topic being discussed in the form of facilitator objectives and prompts (Appendices B and C), a case example (Appendices D and E), and handouts for the learners (Appendices F and G). A member of the study team sent the facilitator information about the patient to be discussed, including identifying information, to allow the facilitator to review the chart prior to the session if they desired. The study team member also provided the facilitator with a short summary of the patient's medical history via email. We did not have formal training sessions prior to implementing the sessions; however, the study team communicated with the facilitator prior to the session to review any questions they might have. A member of the study team was also on hand to provide any support that might be needed during the session.

At the session's start, the facilitator briefly framed the goals of the curriculum, as well as providing an overview of the session. During the first 5 minutes of the session, the trainee presented the patient to be discussed, including the history, reason for admission, and hospital course thus far. The facilitator then spent 10–15 minutes guiding an interactive discussion about the learning objectives with respect to the patient. This portion included multidisciplinary team members, such as dieticians or providers from other specialties related to the topic. Depending on the available time and course of the discussions, the facilitator could choose to focus on some of the objectives, rather than covering all four for the given topic. If applicable, the facilitator then spent 10 minutes with hands-on demonstration, for example, examination of medical devices such as feeding tubes, or handouts (Appendices F and G). Over the next 5–10 minutes, the entire group went to the bedside to review pertinent exam findings or discuss the topic with the patient and/or caregiver. Finally, the group wrapped up by clarifying any final questions, and the learners shared with the group something that they would take away from the session, as well as completing evaluation forms (Appendices H and I).

### Evaluation and Analysis

We developed an evaluation to assess learners’ reactions, learning, and intended behavior change.^17^ At the conclusion of the session, learners received an anonymous evaluation tool, utilizing a retrospective pre/post design, as created by the authors (Appendices H and I). This design was used to reduce response-shift bias, in which the frame of reference for abilities changes on the basis of the intervention rendering pretest scores inaccurate.^18^ Eight items assessed learners’ self-efficacy on the learning objectives using a 5-point scale (1 = *beginner,* 5 = *expert*). Nine items assessed the acceptance of the curriculum using a 4-point scale (1= *strongly agree,* 4 = *strongly disagree*). The literature review had not revealed validated items applicable to this project, so survey items were developed by the study team and piloted for readability with pediatric chief residents at the program. At the end of their inpatient rotation, learners on the other inpatient team who did not receive the complex care curriculum completed an evaluation that assessed the same eight self-efficacy items with respect to the start and end of their rotation. They served as the control group to assess the natural skill acquisition that occurred through clinical experiences on the inpatient service.

The evaluation form also featured open-ended questions about how the learner's practice might change after participating in the session, as well as suggestions for improvement. Additionally, a subset of five participants was recruited for brief focused interviews with an individual (Ashley Croker-Benn) not affiliated with the residency program to gather data regarding sustained change in behavior 6 months following receipt of the CMC sessions.

We distributed our surveys over 12 months, May 2021-May 2022. We used descriptive statistics to describe reactions and to calculate mean differences in pre- and postintervention ratings, paired *t* tests to analyze changes in self-efficacy ratings within groups, and independent *t* tests to compare mean differences between the participants who received the intervention and those who received the control.

This project was reviewed by the Columbia University Institutional Review Board and determined to be exempt.

## Results

Over the 12-month study period, students and residents participated in educational sessions every 2 weeks with focus alternating between the two topics. In total, 72 evaluations of the feeding/nutrition session and 78 evaluations of the pain/irritability session were completed; number and characteristics of nonrespondents were not tracked. Sessions typically had eight to nine attendees: four interns, two senior residents (PGY 2 or PGY 3), and two to three medical students. Of the intervention group respondents, 36% were first-year residents, 12% second-year residents, 13% third-year residents, and 39% medical students. All participants agreed or strongly agreed that this curriculum was an effective and engaging way to learn, that sessions had the appropriate amount of information, and that they would like to have more sessions in the future. Nearly all participants reported that their knowledge, ability, and confidence in caring for CMC had improved as a result of the session (Table 1).

**Table 1.**
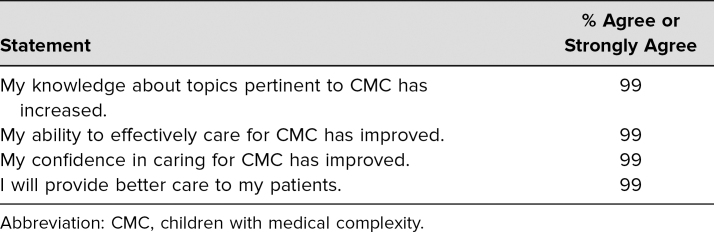
Self-Evaluation After Complex Care Curriculum

The greatest improvements in self-efficacy in those receiving the curriculum were seen in the feeding/nutrition session regarding ability to troubleshoot feeding tube–related problems (mean difference = 0.9), risks and benefits of feeding tube placement (0.9), and knowledge of different feeding tubes (0.8). Participants overall reported statistically significant improvement in self-efficacy for each of the eight items related to achievement of the learning objectives. Subgroup analysis of medical students and residents in different groups demonstrated there was still an improvement in self-efficacy scores from pre- to postintervention for all eight learning objectives (*p* < .001). Participants in the control group completed surveys at the end of the 4-week inpatient rotation and reported improvement in self-efficacy for seven of the eight learning objectives (*p* < .05; Table 2).

**Table 2.**
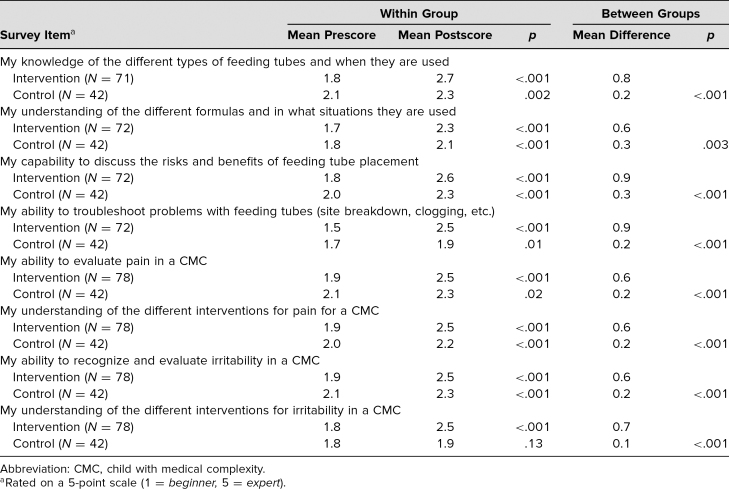
Mean Pre- and Postrotation Self-Efficacy Scores Within Groups and Mean Difference Comparison Between Groups

The intervention group showed statistically greater improvement in self-efficacy in all eight learning objectives when compared with the control group (Table 2).

Free-text survey comments and quotes from five participant interviews conducted at 6 months demonstrated that participants reported positive reaction to the sessions, acquisition of knowledge, and intended change in behavior as a result of the curriculum. Representative quotes are presented in Table 3, sorted by level of Kirkpatrick's model,^17^ along with participants’ suggestions for improvement.

**Table 3.**
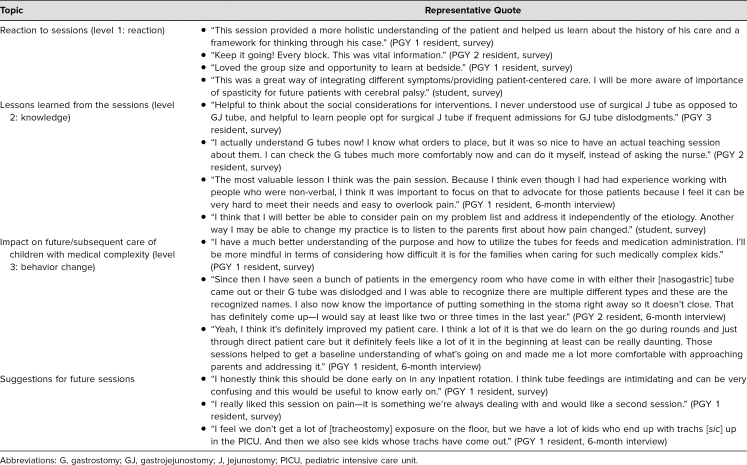
Comments From Written Evaluations and Interviews Mapped to New World Kirkpatrick Levels of Evaluation and Suggestions for Improvement

## Discussion

Implementation of a patient-focused CMC curriculum was met with positive reactions, knowledge acquisition, and subjective reports of changes in behavior when caring for CMC. Our findings suggest that a dedicated curriculum for CMC increases self-efficacy beyond what is gained through the normal progression of clinical rotations, as shown by the greater improvement in self-efficacy scores for the intervention group as compared to the control group, thus establishing the benefit of targeted learning to help learners reinforce and apply clinical principles when caring for this vulnerable population with complex medical needs. By utilizing current patient cases, we were able to utilize higher-level principles of adult learning beyond didactics. Learners’ knowledge and self-efficacy regarding caring for CMC increased in the control group as well, highlighting the important skill acquisition that naturally occurs over time while caring for CMC in an inpatient rotation. Additionally, this was a feasible educational strategy easily incorporated into trainees’ workday.

This curriculum can be adapted and implemented in other pediatric training programs. Though the sessions occupy roughly 45 minutes, the study team envisions that objectives can still be met in less time based on available time, resources, and needs of other programs. The facilitator was often joined by the service attending, who provided additional insight and could utilize this time as rounds for that specific patient, particularly if the group was able to make a bedside visit at the end of the session as intended. At our institution, it was variable amongst our group whether the service attending used the session as the rounding time. Minimal preparatory time was required by the facilitators, and the participants seemed to gain the most from the hands-on portion with the feeding tubes/mannequin and the bedside examination. Though our institution had a complex care team comprising one faculty hospitalist and one nurse practitioner, many sessions were led by other hospitalists and harnessed the expertise of existing health care providers, including registered dieticians, nurses, and pediatric gastroenterology fellows and faculty, thus also modeling competencies around interdisciplinary care and collaboration.

Recently proposed entrustable professional activities (EPAs) for the care of CMC supports the need for dedicated educational time and additional curriculum development for pediatric trainees around this topic.^19^ These proposed EPAs and their mapping to ACGME milestones can be incorporated into the formal evaluation of pediatric residents to further assess their performance following participation in a curriculum such as ours.

There are some limitations to the generalizability of our findings. Curriculum evaluations centered on self-efficacy data without patient outcome-level data. The results could also reflect social desirability bias, with respondents reporting the level of self-efficacy they thought would be viewed favorably, rather than a true evaluation of their knowledge. Given that residents rotated through both inpatient teams over the course of the academic year, the difference between the intervention and control groups may have been underestimated due to crossover between groups. Facilitators were instructed that they need not cover every learning objective during the session, depending on the direction of the discussion, available time/competing clinical demands, and the clinical details of the patient being discussed. Not covering every learning objective could also have impacted the results, though one would expect this to bias toward the null. Our institution previously had another teaching session in this time slot and therefore did not require a major shift in timing for our program. This may present a challenge to other institutions, which may need to create a morning time slot for this type of rounds-adjacent clinical curriculum. Finally, this curriculum may require a greater time commitment from facilitators as it occurs on a recurring basis, rather than being a onetime lecture. However, the repetition likely contributes to reinforcement and retention of learners’ knowledge and skills.

Future steps include expanding this model to other topics related to CMC. We initially started the curriculum with the two topics of the greatest need at our institution based on a targeted needs assessment. Since implementation, due to positive learner and facilitator feedback, we have started to expand the curriculum to include sessions on spasticity management, airway clearance, emergency care plans, dysmotility, and ventriculoperitoneal shunts. We evaluated the curriculum at New World Kirkpatrick levels 1, 2, and 3 (reaction, learning, and behavior change), as this version of the Kirkpatrick levels allowed for self-report of knowledge and behavior change to reflect evaluation of levels 2 and 3.^17^ Next steps may focus on implementing higher-level evaluation of the curriculum, including direct faculty observations to assess resident competency in caring for CMC and observed simulated encounters to assess scenarios such as a malfunctioning feeding tube and acute pain.

In summary, this curriculum showed a significant improvement in learner-reported self-efficacy in caring for CMC when compared to the natural learning process occurring during an inpatient rotation. We believe the curriculum can be adopted by other training programs and integrated into their academic learning time. By anchoring the sessions in the clinical setting, the trainees learn through the experience of caring for these patients, with the facilitator serving as a guide to supplement their learning. As medicine and technology advance, this patient population will continue to grow and account for more health care utilization, emphasizing the need for more dedicated trainee education.

## Appendices


General Facilitator Guide.docxFeeding Nutrition Facilitator Objectives and Prompts.docxPain Irritability Facilitator Objectives and Prompts.docxFeeding Nutrition Case Example.docxPain Irritability Case Example.docxFeeding Nutrition Handout.docxPain Irritability Handout.docxFeeding Nutrition Evaluation.docxPain Irritability Evaluation.docx
*All appendices are peer reviewed as integral parts of the Original Publication.*
