# Supplementary material for: Caring for Children With Medical Complexity: A Clinical, Patient-Focused Curriculum
Source: MedEdPORTAL. 2024 Jan 30;20:11380. doi: 10.15766/mep_2374-8265.11380 (PMC10825041; doi:10.15766/mep_2374-8265.11380)
Supplement: Supplementary file 1 — General Facilitator Guide.docxFeeding Nutrition Facilitator Objectives and Prompts.docxPain Irritability Facilitator Objectives and Prompts.docxFeeding Nutrition Case Example.docxPain Irritability Case Example.docxFeeding Nutrition Handout.docxPain Irritability Handout.docxFeeding Nutrition Evaluation.docxPain Irritability Evaluation.docx [file mep_2374-8265.11380-s001.zip › A. General Facilitator Guide.docx]

CMC Session General Facilitator’s Guide

This guide is intended for facilitators to use in preparation for the session. It should be reviewed prior to the session to give an overview of the different components and how they should be divided over the 45-minute session. This guide can also serve as a reference during the session to keep appropriate timing.

Prior to the session, the curriculum team should:

- Identify content facilitator(s) for session
- Identify a patient to discuss during the session
  - Patient should:
    - meet criteria for being medically complex (having chronic conditions, functional limitations often dependent on technology, high health care utilization, and substantial family identified needs)
    - be primarily cared for by the learners on the inpatient team
    - have either acute or chronic problems that are relevant to the topic being discussed
    - ideally still be hospitalized so that the discussion is timely and relevant. This also allows for the possibility of going to the bedside for demonstration and application.
- Identify a space that allows group to gather and have a discussion with minimal interruptions. It is also important to allow for privacy and confidentiality as a patient will be discussed.

Prior to the session, the facilitator should:

- Review this Facilitator’s Guide to understand the outline of the session, and bring to the session as a reference
- Review the Objectives and Suggested Prompts for the pertinent session (Appendices B and C), and bring to the session as a reference
- Review the Sample Case for the pertinent session (Appendices D and E)
- Review the Handout for the pertinent session (Appendices F and G), and bring to the session if the facilitator intends on using it during the session
- If pertinent to the session, bring any medical equipment that might be used (e.g., sample feeding tubes)
- Bring surveys to the session if there is an intention to distribute them at the end (Appendices H and I)

Session Outline (total 45 minutes)

1. Introduction and objectives (2 minutes)
   1. All participants and facilitators should introduce themselves to the group and state their role (medical student, dietitian, etc.)
   2. Facilitator can briefly frame the session for the group.
      1. They may explain how the goal of the session is to learn about feeding/nutrition or pain/irritability in children with medical complexity (CMC) by focusing on a patient the team is already caring for. They can also provide a brief breakdown of the components of the session as detailed below.
2. Present patient history (approximately 5 minutes)
   1. The learner (resident or medical student) who is the primary provider for the patient will present the patient to the learning group.
   2. Learner should present a brief overview of the patient’s medical history, reason for hospital admission, hospital course thus far, and ongoing problems to be addressed during the admission.
3. Facilitated discussion (10-15 minutes)
   1. Facilitator will lead an interactive discussion about the patient, focusing on the learning objectives (Appendices B and C)
      1. The goal is to cover at least 1-2 objectives that are most relevant to the patient and their hospitalization. Facilitators may not cover all four objectives for each session depending on the time and course of discussion.
   2. The discussion should be centered on the patient being discussed. Facilitators should use the patient’s history, medications, feeding regimen, etc. as a way to discuss the topic.
   3. Learners should be encouraged to ask questions to allow for a discussion of the topic, rather than a lecture by the facilitator(s).
   4. Facilitator(s) can use the additional prompts provided (Appendices B and C) to help guide a discussion that encourages all group members to participate. This is not meant to be a prepared didactic presentation.
4. Hands-on demonstration, when applicable (10 minutes)
   1. Utilize expertise of any invited guests such as allied health professionals and subspecialists to review basics of caring for CMC. Examples include: calculate a feeding rate for tube feeds based on caloric needs, demonstrate how to inflate a gastrostomy tube balloon on a mannequin, review how to rate pain using a scale. What the facilitator chooses to discuss during this portion may vary based on the discussion that the group is having, and what the expertise is of any additional facilitators. The above examples provide suggestions that may or may not all be included during the session.
   2. Facilitator(s) may bring materials, such as different feeding tubes, or handouts demonstrating different pain evaluation scales
   3. Learners may use prepared handouts (Appendices F and G) as aids.
5. Bedside portion/application (5-10 minutes)
   1. If feasible, the facilitator should bring the learners to the bedside to continue the discussion with the patient and/or caregiver directly.
   2. The facilitator(s) may demonstrate pertinent physical exam findings, walk through the components of the patient’s medical technology/equipment, or lead a discussion with the patient and/or caregiver at bedside.
6. Wrap-up (3-5 minutes)
   1. The group should gather and summarize major learning points from the discussion. Learners can individually share a main take-away from the session with the group.
   2. Any final questions should be answered.
7. Conclusion
   1. Facilitator(s) distribute anonymous surveys in order to assess the efficacy of the session (Appendices H and I).
