# Supplementary material for: Caring for Children With Medical Complexity: A Clinical, Patient-Focused Curriculum
Source: MedEdPORTAL. 2024 Jan 30;20:11380. doi: 10.15766/mep_2374-8265.11380 (PMC10825041; doi:10.15766/mep_2374-8265.11380)
Supplement: Supplementary file 1 — General Facilitator Guide.docxFeeding Nutrition Facilitator Objectives and Prompts.docxPain Irritability Facilitator Objectives and Prompts.docxFeeding Nutrition Case Example.docxPain Irritability Case Example.docxFeeding Nutrition Handout.docxPain Irritability Handout.docxFeeding Nutrition Evaluation.docxPain Irritability Evaluation.docx [file mep_2374-8265.11380-s001.zip › B. Feeding Nutrition Facilitator Objectives and Prompts.docx]

Children with Medical Complexity (CMC) Curriculum: Feeding/Nutrition Session

Learning Objectives, Facilitator Prompts/Suggestions

This guide is intended for facilitators to use in preparation for a feeding/nutrition session. It should be reviewed prior to the session so that the facilitator knows the objectives of the session, and potential questions/prompts to use for guiding the interactive discussion about the patient. It can also serve as a reference during the session to help facilitate the discussion.

**Learning Objectives:***

At the end of the session, learners should be able to:

1. Identify different types of enteric feeding tubes and describe the clinical scenarios in which they are used.
2. List the different types of formulas and describe the clinical scenarios in which they are used.
3. Discuss risks and benefits of feeding tube placement.
4. Identify steps to troubleshoot common problems associated with feeding tubes.

** Depending on time and the course of the discussion, facilitators may choose to focus on some of the objectives, rather than covering all four*

**The facilitator may use the following as suggestions to guide an interactive discussion about the patient being discussed. It is not the intention or goal to use all of the following suggestions, and facilitators may use other questions/prompts to guide the discussion based on their own knowledge and expertise.**

- What are the indications for this patient to have a feeding tube?
  - Facilitators should use this as a chance to discuss the specific patient and their reason for a feeding tube. They can then expand the discussion to include other indications for feeding tubes. Examples include insufficient oral intake in setting of malabsorption (cystic fibrosis, short bowel syndrome, etc.), increased nutritional needs (congenital heart disease), as a means of delivering treatment in metabolic disease, any oral motor dysfunction or swallowing dysfunction. Appendix F provides a list of some indications for tube feeding which may be useful for the facilitator to bring to the session for the learners.
- What type of feeding tube does the patient have? Why does the patient have this type of feeding tube? (e.g., why a G tube instead of a GJ?)
  - Facilitators can use this as an opportunity to broadly talk about the differences between types of feeding tubes and why you would choose one over the other. They may bring Appendix F with them to the session, which has an outline of the different types of feeding tubes and some distinguishing features.
  - Facilitators may consider bringing feeding tubes for learners to examine and look at. Demonstrate how to determine the size of the tube, how to inflate/deflate the balloon.
- Is this patient receiving other types of nutrition, such as oral or parenteral? Discuss the role of each form of nutrition in this patient.
  - If the patient is also receiving parenteral nutrition, things to discuss with the group may be what about this patient’s GI tract is preventing adequate nutrient delivery and absorption through the enteral route. Perhaps there is a GI obstruction or intestinal ischemia. If they are receiving parenteral nutrition, the facilitator may discuss how, if possible, the enteral route is preferred because it can lead to earlier gut function, lower infections (no need for central access), shorter hospital stays, and lower health-care costs.^20^ If the patient is receiving some form of oral nutrition, why is that? Is this a way to maintain the patient’s oral motor skills, but the amount of oral nutrition is inadequate for daily nutritional needs?
- How would you present the need for feeding tube to this patient and their family?
  - This may be a chance for the facilitator to have members of the group to practice or role-play presenting the need for feeding tube to a patient and/or their family member. The learner may include discussing why the specific patient needs a feeding tube, what the consequences are if a feeding tube is not placed (this may include malnutrition, poor growth, certain vitamin deficiencies), the types of tubes that may be indicated for the patient (is this an acute or chronic need for feeding tube), what the risks/complications are of feeding tubes. The facilitator can help guide the group in using empathetic communication skills. This is also an opportunity to review that the placement of a feeding tube can be very stressful and frightening for family members. The facilitator may draw on their own experience of having to discuss placement of a feeding tube for previous patients and provide personal anecdotes of what has and has not worked for them in those situations.
- What are the risks of placing a feeding tube that should be discussed with the family?
  - The risks of placing feeding tube will vary based on the type of tube placed. For more chronic tubes, like G or J tubes, there is the risk of anesthesia as these are placed under sedation. Immediately post-procedure there could be the risk of misplacement, bleeding, infection. There may be some pain post procedure as well. For short-term feeding tubes, like NG tube there may be discomfort with placement, misplacement requiring multiple insertions.
- Has this patient had issues with their feeding tube during this admission or outside of the hospital? How might you address them?
  - This is a chance for the facilitator to review the patient’s history both during this admission, and prior if it is known to the team. If the team does not know about prior issues with the patient’s feeding tube, this may be a question that could be asked to the patient/family during the bedside portion of the session (as indicated below).
- What are common issues or challenges associated with feeding tubes? How might you instruct a family member to troubleshoot these at home?
  - Common issues include the tube becoming dislodged or removed. Depending on the type of tube and the family’s training, they might be able to replace the tube at home on their own. For example, families may have been trained on how to replace a G tube at home, so they can replace a dislodged tube on their own. GJ tubes, however, cannot be replaced by the family so they must present to medical attention. Other complications include leaking around the site. Families should assess that the balloon is correctly inflated.
- Facilitators may consider reviewing the patient’s current feeding regimen with the team. Why are they on this particular formula?
  - Facilitators may use this as a chance to review broad categories of formulas. Singhal, et al summarize different categories of formulas that may be helpful for the facilitator to review and use during the session.^20^ Categories include standard pediatric enteral formulas, calorie-dense pediatric formulas, reduced-calorie pediatric enteral formulas, hydrolyzed pediatric formulas, free amino acid pediatric formulas. Facilitators may discuss which category the patient’s formula falls into and why it was indicated in this patient.
- Facilitators may discuss how to determine the patient’s goal caloric intake, free water needs, or other nutritional needs.
  - This may be an instance where the facilitator may want to partner with a member of the nutrition or gastroenterology team. Appendix F provides an example calculation that facilitators may use with the group to demonstrate how to determine nutritional needs.^21, 22^
- Facilitators may discuss how feeding regimens differ for different types of tubes (continuous vs. bolus)?
  - Bolus feeds are generally given for gastric feedings and not for postpyloric feedings as the stomach can accommodate large volumes. GJ or J tube feedings are generally continuous feeds which are a constant hourly rate over a longer period of time. Some patients have a combination of bolus and continuous feeds.
  - This is a chance to review the patient’s feeding orders and discuss why they have the regimen that they have.
- How was the patient’s feeding rate determined?
  - After discussing the goal total daily volume of formula that the patient needs and whether bolus or continuous feeds are indicated, the facilitator may review how the feeding rate was determined. They can discuss factors that go into determining the feeding rate. This may include family preference for feeds. For example, a family may prefer to have 3 bolus feeds during the day and one overnight continuous feed for ease with their schedule, and the facilitator can discuss how the team might distribute the total daily formula over those feedings. Singhal, et al also provide an overview of goal feeding rates for the different types of feeds (continuous vs. bolus), which may be helpful for the facilitator to review both prior to the session and with the learners.^20^
- Is this patient receiving postpyloric feedings and why?
  - Postpyloric feeding is when nutrition is delivered beyond the pylorus, into the duodenum or jejunum. General indications for postpyloric feedings include patients at risk for aspiration, intolerance to gastric feeding, recurrent emesis, severe gastroesophageal reflux. The facilitator should review the patient’s orders and discuss their specific indication for postpyloric feedings if applicable.
- Is the patient on any supplements or vitamins? What are the indications for them?
  - This is a chance for the facilitator to review the chart/medication list with the group. As a group they may be able to review prior labs or notes that detail why the patient is on certain supplements. If there are members of the nutrition or gastroenterology team participating in the session, they may also serve as a resource to help answer these questions.
- If possible, facilitators may bring a copy of the patient’s growth chart to discuss target growth, weight, height. Does the patient have a condition that has a specific growth chart that is different from the general pediatric growth chart?

**At the bedside:**

The facilitator has a variety of options when approaching the bedside portion of the session. They can use this as a time to review certain exam findings that relate to the discussion that was had, examine medical technology that is pertinent to the topic, practice asking the patient/caregivers questions that had been discussed (a way for learners to practice communication skills), or give the patient/caregiver a chance to express their own experiences and knowledge they wish their providers had.

Some potential suggestions the facilitator may consider at the bedside:

- Examine the patient’s feeding tube and discuss different components (e.g., pump, extension tubing, etc.). Demonstrate concepts such as how to attach/remove tubing, determine the size of the tube, or inflate/deflate balloon. This would require the patient to agree to this examination, and may be repetitive if this was already discussed/demonstrated during the hands-on portion of the session.
- If applicable, examine the skin around the feeding tube. Discuss what patients and their caregivers may do to minimize skin irritation. This could be a chance for the facilitator to again review complications that occur with feeding tubes, and a chance for the patient/caregiver to discuss their own experience.
- Discuss with the patient and/or caregiver their experience with having a feeding tube. Potential questions to ask them include:
  - How was the decision made to place a feeding tube?
  - What were their emotions around placing a feeding tube?
  - What have been challenges with having a feeding tube?
  - Have they tried different formulas? Why have they switched and what was that experience like?
  - What are things they have to commonly troubleshoot at home?
  - Are there things they wished they had known prior to the feeding tube being placed?

**For further reference**:

1. Singhal S, Baker SS, Bojczuk GA, Baker RD. Tube feeding in children. *Pediatr Rev*. 2017;38(1):23-34. <https://doi.org/10.1542/pir.2016-0096>
2. Otten JJ, Hellwig JP, Meyers LD, eds. *DRI, Dietary Reference Intakes: The Essential Guide to Nutrient Requirements*. National Academies Press; 2006.
3. Holliday MA, Segar WE. The maintenance need for water in parenteral fluid therapy. *Pediatrics*. 1957;19(5):823-832.
