# Supplementary material for: Caring for Children With Medical Complexity: A Clinical, Patient-Focused Curriculum
Source: MedEdPORTAL. 2024 Jan 30;20:11380. doi: 10.15766/mep_2374-8265.11380 (PMC10825041; doi:10.15766/mep_2374-8265.11380)
Supplement: Supplementary file 1 — General Facilitator Guide.docxFeeding Nutrition Facilitator Objectives and Prompts.docxPain Irritability Facilitator Objectives and Prompts.docxFeeding Nutrition Case Example.docxPain Irritability Case Example.docxFeeding Nutrition Handout.docxPain Irritability Handout.docxFeeding Nutrition Evaluation.docxPain Irritability Evaluation.docx [file mep_2374-8265.11380-s001.zip › C. Pain Irritability Facilitator Objectives and Prompts.docx]

Children with Medical Complexity (CMC) Curriculum: Pain/Irritability Session

Learning Objectives, Facilitator Prompts/Suggestions

This guide is intended for facilitators to use in preparation for a pain/irritability session. It should be reviewed prior to the session so that the facilitator knows the objectives of the session, and potential questions/prompts to use for guiding the interactive discussion about the patient. It can also serve as a reference during the session to help facilitate the discussion.

**Learning Objectives***

At the end of the session, learners should be able to:

1. Outline components of the history and physical examination that aid in evaluating pain in a child with medical complexity (CMC).
2. List different pharmacologic and nonpharmacologic interventions for pain in CMC.
3. Outline components of the history and physical examination that aid in recognizing and evaluating irritability in CMC.
4. List different pharmacologic and nonpharmacologic interventions for irritability in CMC.

** Depending on time and the course of the discussion, facilitators may choose to focus on some of the objectives, rather than covering all four*

**The facilitator may use the following as suggestions to guide an interactive discussion about the patient being discussed. It is not the intention or goal to use all of the following suggestions, and facilitators may use other questions/prompts to guide the discussion based on their own knowledge and expertise.**

**Of note, it is important to distinguish between CMC and patients with severe neurologic impairment (SNI). CMC does not necessarily mean there is impairment in cognitive or communication skills. SNI is defined by having impairment of the central nervous system that results in lifelong intellectual disability and limited verbal communication.^23^ Some of the following suggested prompts/questions may only apply to those with SNI, and not necessarily all patients characterized as having medical complexity.**

- What questions might you ask the patient and/or caregiver in order to evaluate the patient’s pain and/or irritability?
  - To promote an interactive discussion, the facilitator may ask the group to provide questions they have asked families or patients in the past to assess pain and/or irritability. The facilitator may want to discuss using the family/caregiver as a resource to understand the child’s baseline behaviors and what changes they have noticed in the past when there is discomfort.
  - This may be an opportunity to discuss how evaluation changes based on the patient’s neurologic status and ability to communicate. This is especially the time to partner with others who know the patient well. They may ask: how do you know when the patient is in discomfort, is this their usual behavior or are they acting differently now, what has typically caused the patient to have pain in the past (this question may get at etiology of the pain, such as constipation).
- If the team has described the patient as “irritable” and/or “agitated,” the facilitator may ask the group what does that mean to them when they use those words? The facilitator may probe into what aspects of the physical exam (vital signs, general appearance, specific exam systems) that lead the team to use those descriptors.
  - This is an opportunity for the facilitator to review the definitions of irritability and agitation. Hauer, et al, provide definitions for these terms. Agitation is an “unpleasant state of arousal manifesting as irritability, restlessness, and increased motor activity.” Irritability is described as “a disorder characterized by an abnormal responsiveness to stimuli or physiologic arousal.” The facilitator may want to review that the language used is important, because using terms like agitation or irritability may take the focus away from pain, and therefore not treat a child’s pain adequately. Agitation and irritability may indicate an irritable nervous system, and therefore guide the team away from using medications directed at pain etiologies.^23^
- What are the different types of pain? What type of pain may this patient have?
  - Examples: nociceptive, neuropathic, allodynia, hyperalgesia. The facilitator can refer to Appendix G for definitions to review with the group. After reviewing the different types of pain, the group may discuss what type of pain they think their patient might have. For example, did the patient just recently have a procedure performed? This may indicate that they have nociceptive pain.^23, 24^
- If the patient has seizures or a movement disorder, what might questions might you ask to find out their semiology?
  - The facilitator may ask the group to provide sample questions that they would ask families about the patient’s seizure semiology or movement disorders.
  - Examples include: can you describe what their seizures typically look like? Is their current movement typical for them or is it different? Do you notice that their movements change in response to certain triggers?
- What do the words tone, dystonia, spasticity, and spasms mean?
  - The facilitator may consider differentiating between these words as they may often be used incorrectly when describing a patient’s exam. Hauer, et al, provides definitions the facilitator may want to review and use during the discussion. They are provided here for convenience.^23^ These terms are also defined in Appendix G, which the facilitator can use during the session.
  - Tone is a stiffening of the extremities, clenching of fists, back arching, and resists movement
  - Dystonia is involuntary sustained or intermittent muscle contractions cause twisting and repetitive movements, abnormal postures, or both
  - Spasticity is velocity-dependent increase in muscle tone that results in muscles that are resistant to movement
  - Muscle spasms are sudden involuntary contraction of a muscle or group of muscles
- If the patient has technology, what is their typical response to treatments (for example if they use chest vest, cough assist, CPAP, etc.). Does the patient’s behavior change during these treatments?
  - The facilitator and group may want to discuss whether the patient exhibits more pain behaviors during the treatment that then resolve afterwards
- What pain behaviors does the patient exhibit?
  - Learners may discuss what they have observed the patient doing, discuss how they would ask a caregiver about pain behaviors, and practice asking at the bedside.
  - Hauer, et al, provide an overview of different pain behaviors that the facilitator may want to review and discuss with the group. These behaviors include vocalizations (crying, moaning), facial expression (grimacing, teeth grinding), consolability, interaction (withdrawn, seeking comfort), sleep (increased or decreased sleep), movement (increased from baseline, restless), tone (stiffening of extremities), physiologic signs (tachycardia, sweating, change in color), atypical features (blunted facial expression, self-injurious behaviors).^23^
- Why might this patient have pain or be irritable?
  - Group should discuss patient disease specific etiologies as well as common pediatric causes. Hauer, et al, provides a list of potential nociceptive etiologies for pain behaviors. These include corneal abrasion, dental caries, ventriculoperitoneal shunt malfunction, constipation, hair tourniquet, pressure ulcer, occult fracture, subluxation (especially of the hips), urinary tract infection.^23^ Perhaps this patient has neuropathic pain, as indicated by descriptors such as burning, shock-like, shooting. They may describe the pain as persistent or recurrent. Other words used in the description of neuropathic pain include allodynia (pain from a stimulus that does not normally result in pain) or hyperalgesia (increased pain to a painful stimulus). Some etiologies include injury or disease of the CNS involving the thalamus or spinothalamic tract.
- The facilitator may consider reviewing the patient’s medication list. Depending on the facilitator’s comfort and familiarity with the medications, this may or may not require some review prior to the session. If the team has a clinical pharmacist available, they may be an additional member the team may want to consider inviting to the session. Finally, Appendix G provides a list of the major pharmacologic categories of medications used in pain and irritability.^23,^ ^25^ The facilitator can use during the session to review with the group. The facilitator may discuss:
  - the indication for each medication in the specific patient being discussed
  - the unintended benefits or side effects of the medications
  - risks and signs of withdrawal of these medications, if applicable
  - risks and signs of overdose of these medications, if applicable
- Discuss different pain scales that have been validated for use in evaluating pain. The facilitator may consider bringing copies of the scales and assessing the patient at the bedside using it.
  - There are numerous scales to consider.
  - Tools for children with SNI include:
    - Revised FLACC (Appendix G)^26^
    - Individualized Numerical Rating Scale (INRS) (Appendix G)^27^
    - Others you may consider using include the Non-Communicating Children’s Pain Checklist-Revised (NCCPC-R), Pediatric Pain Profile (PPP). This will require the facilitator to review and bring to the session on their own.
- Are there any other explanations for the patient’s pain behaviors that might not be pain related?
  - The facilitator may review the following examples as other reasons why the patient may be exhibiting pain behaviors. These may include medication toxicity and withdrawal, delirium, autonomic dysfunction, seizures, spasticity, movement disorders, etc. Is there anything on the patient’s history that may indicate these. Perhaps they recently had a change in dose to their medication which may now be leading to withdrawal. Were they in the ICU for an extended period of time on sedating drugs and may now be exhibiting delirium?
  - It is important to remember that increased tone and movement may be as a result of spasticity and dystonia, but can also be an indication of increased pain. Perhaps the team has been focusing on treating the spasticity with little success, and this may be a forum to discuss that maybe the patient needs treatment directed at pain. The facilitator may point out that in children with SNI, it can often be difficult to determine the etiology of certain pain behaviors, and it is important to keep a broad differential rather than anchoring on a cause.
- What are major categories of pain medications? Which might be helpful to use for this patient? Are there any that are contraindicated in this patient? Is there a role for these medications to help treat their irritability? If the patient has already been prescribed a medication, habitually checking if the dosage needs to be weight adjusted may be important. What category of medications would you start with and what might be a second line agent?
  - The facilitator may use Appendix G as a guide to review the major categories of pain medications. By reviewing the patient’s current medication orders, the group can determine which category the specific medication belongs to, and what are the potential risks of the medication.
- What else needs to be considered when prescribing pain medications?
  - This is a chance for the facilitator to emphasize the chronic level of care that parents and caregivers need to provide for CMC. Facilitators can bridge the medical discussion with aspects of the patient’s life that providers should consider when recommending interventions.
  - The facilitator can consider discussing the following:
    - Dosing intervals: does this child need around the clock administration or as needed administration? Does this schedule work for the child and/or family’s other activities?
    - Route of administration: is oral adequate or are there other routes of administration needed?
- Are there adjuvant medications that could be used for this patient?
  - Examples: anticonvulsants, antidepressants, lidocaine patches, anti-constipation medications, etc.
- What are non-pharmacologic interventions that could be used in this patient?
  - Examples: repositioning, braces, stretching, massage, warm baths, hot/ice packs, swaddling, rocking, supportive equipment, music, integrative therapies, gastrostomy tube venting, etc.
  - The facilitator can refer to Appendix G for a list to review with the group. It is important to discuss these interventions, because pharmacologic interventions may have limited efficacy, intolerable side effects, or medical contraindications. Non-pharmacologic interventions provide the family/caregiver with a holistic approach to managing the child’s discomfort.

**At the bedside:**

The facilitator has a variety of options when approaching the bedside portion of the session. They can use this as a time to review certain exam findings that relate to the discussion that was had, examine medical technology that is pertinent to the topic, practice asking the patient/caregivers questions that had been discussed (a way for learners to practice communication skills), or give the patient/caregiver a chance to express their own experiences and knowledge they wish their providers had.

Some potential suggestions the facilitator may consider at the bedside:

- Engage in a discussion with the patient and/or their caregiver about how they recognize the patient is in pain. This could be a chance for the learners to practice asking the questions they had discussed earlier in the session.
- Practice non-judgmental conversations about the adherence to therapies. The learners may want to practice asking the patient/caregiver about adherence, and practice empathetic responses. This can allow the group to have a greater appreciation for why patients might not be adherent to medications.
- Discuss what has and has not worked in the past to treat these symptoms.
- Discuss what has been a common source of the pain and/or irritability in the past.
- Examine the patient when applicable (e.g., if the patient has significant spasticity, discuss tone assessment, velocity dependence, changes in reflexes, contractures and weakness, and emphasize the difference between dystonia/rigidity/spasticity). The physical exam portion will depend on the facilitator’s own comfort and expertise in doing these specific physical exam maneuvers.

**For further reference:**

1. Hauer J, Houtrow AJ; Section on Hospice and Palliative Medicine Council on Children With Disabilities. Pain assessment and treatment in children with significant impairment of the central nervous system. *Pediatrics*. 2017;139(6):e20171002. <https://doi.org/10.1542/peds.2017-1002>
2. Terminology. International Association for the Study of Pain. Accessed October 19, 2022. <https://www.iasp-pain.org/resources/terminology/>
3. Giordano M, Knipper E, Melwani A. Clinical progress note: perioperative pain control in hospitalized pediatric patients. *J Hosp Med*. 2021;16(6):358-360. <https://doi.org/10.12788/jhm.3388>
4. Malviya S, Voepel-Lewis T, Burke C, Merkel S, Tait AR. The revised FLACC observational pain tool: improved reliability and validity for pain assessment in children with cognitive impairment. *Pediatr Anesth*. 2006;16(3):258-265. <https://doi.org/10.1111/j.1460-9592.2005.01773.x>
5. Solodiuk JC, Scott-Sutherland J, Meyers M, et al. Validation of the Individualized Numeric Rating Scale (INRS): a pain assessment tool for nonverbal children with intellectual disability. *Pain*. 2010;150(2):231-236. <https://doi.org/10.1016/j.pain.2010.03.016>
