# Supplementary material for: Caring for Children With Medical Complexity: A Clinical, Patient-Focused Curriculum
Source: MedEdPORTAL. 2024 Jan 30;20:11380. doi: 10.15766/mep_2374-8265.11380 (PMC10825041; doi:10.15766/mep_2374-8265.11380)
Supplement: Supplementary file 1 — General Facilitator Guide.docxFeeding Nutrition Facilitator Objectives and Prompts.docxPain Irritability Facilitator Objectives and Prompts.docxFeeding Nutrition Case Example.docxPain Irritability Case Example.docxFeeding Nutrition Handout.docxPain Irritability Handout.docxFeeding Nutrition Evaluation.docxPain Irritability Evaluation.docx [file mep_2374-8265.11380-s001.zip › D. Feeding Nutrition Case Example.docx]

An Example Case of How to Discuss Feeding/Nutrition in CMC

The following is meant to serve as an example of how a learning session may run. We have provided an example patient case, example questions/prompts the facilitator may use to guide the session, and potential responses/discussion points that the facilitator would hope to elicit from the group. The following is to serve as a model of how a session might run, knowing that there are numerous ways the facilitator could use the patient case to discuss the following learning objectives.

**Learning Objectives*:**

By the end of this activity, learners will be able to:

1. Identify different types of enteric feeding tubes and describe the clinical scenarios in which they are used.
2. List the different types of formulas and describe the clinical scenarios in which they are used.
3. Discuss risks and benefits of feeding tube placement.
4. Identify steps to troubleshoot common problems associated with feeding tubes.

** Depending on time and the course of the discussion, facilitators may choose to focus on some of the objectives, rather than covering all four*

The Patient Case:

After group introductions (2 minutes), the facilitator should ask one of the trainees to present the patient to the group, focusing on their past medical history, reason for admission, and hospital course thus far (approximately 5 minutes).

*Trainee: Our patient is a 5-year-old ex-28-week female, with a history of developmental delay, hypotonia, dysphagia, and g-tube dependence, currently admitted for management of aspiration pneumonia on IV antibiotics. She had fever and vomiting for 3 days at home prior to coming to the Emergency Room. Her initial exam was concerning for tachypnea, retractions, and crackles on the right lower lung field. Chest X-ray was concerning for pneumonia. This is her third admission for presumed aspiration pneumonia over the last two years. She had a g-tube placed when she was 2 years old due to dysphagia as diagnosed on swallow study. During this admission her feeds were initially held since she was still having emesis, and she was placed on IV fluids. Today we have started to resume her feeds as she has clinically improved and the emesis has stopped.*

Facilitated Discussion:

The facilitator will now lead an interactive discussion to try and discuss the learning objectives as they relate to the patient case. If possible, these sessions should be multidisciplinary and include members of nutrition, gastroenterology, or any other departments that care for the nutritional needs of pediatric patients. The following is an example of how the facilitator may guide the discussion, with the respective learning objectives in parentheses. This should take approximately 10-15 minutes.

**Facilitator: Our patient has had a g-tube for many years, what were the indications for her to have a g-tube placed? (Learning Objective #1)**

*Trainee: She has dysphagia as diagnosed on a swallow study so she is not cleared to take food by mouth. She needs a tube for nutrition. Her developmental delay and hypotonia may contribute to her dysphagia.*

**Facilitator**: **Exactly. There are many indications for feeding tube placement. These include: insufficient oral intake such as when there is oral aversion, malabsorption like in cystic fibrosis, increased caloric needs like in congenital heart disease; as a primary therapy for a metabolic disease, intolerance to fasting; oral motor dysfunction like in neuromuscular diseases; abnormal gastrointestinal tract like in esophageal stenosis; or in injury/critical illness like burns or trauma. Not every patient has the same indication for tube placement. Some patients will need a feeding tube long-term, whereas others need it for a short-term indication. Our patient has a g-tube, but there are other types of feeding tubes. Can someone describe the difference between a g-tube and a G-J tube?**

*Trainee: A g-tube ends in the stomach, while a G-J tube has a portion that ends in the stomach and a second portion that goes post-pyloric into the jejunum.*

**Facilitator: That is right, is there a reason why we might consider a G-J tube in the future for this patient?**

*Trainee: A G-J may limit the risk of aspiration since feeds would then be post-pyloric. Since she has had multiple aspiration pneumonias in the past, this might be something to consider.*

**Facilitator: Yes, I agree. I have brought with me different feeding tubes. We will pass them around and review the components of it. Does anyone know what the numbers on the tube mean?**

*Trainee*: *The “Fr” is the diameter or the “French.” The other number is the length of the tube which is measured in centimeters.*

**Facilitator**: **Those are important to know in case the tube needs to be replaced, you will need to know what size it is. We will practice manipulating the tubes, such as inflating/deflating the balloon, opening/closing the ports.**

The facilitator can then spend time pointing out the differences between the different feeding tubes and how to use them.

**Facilitator: Now that we have reviewed some different types of feeding tubes, let’s talk about our patient’s g- tube feeding regimen. She currently receives bolus feeds of Pediasure 1.0, 3 times during the day, and continuous feeds overnight. What is the difference between a bolus and a continuous feed? (Learning Objective #2)**

*Trainee: A bolus feed is a large volume of feed (formula) given over a short period of time. It mimics having a meal. A continuous feed is a slower rate of feed that occurs over a longer period of time.*

**Facilitator: We discussed how this patient might benefit from converting the G to a G-J tube in the future. Would her feeding regimen have to change?**

*Trainee: You cannot do bolus feeds when feeding post-pyloric because the intestine cannot accommodate large volumes at once like the stomach can. Our patient would need to be on continuous feeds instead.*

Hands-on Demonstration:

At this point in the discussion, if you have a member from another team such as nutrition or gastroenterology, they may want to review how to calculate the caloric needs of the patient or the free water requirement. They can use the Handout (Appendix F) to help demonstrate the calculations. A facilitator may want to review the patient’s growth chart with the group and review how to assess whether there is appropriate growth or if there is malnutrition. The facilitator may bring other materials, such as different feeding tubes, to use during this portion.

Depending on time, the facilitator(s) could use additional suggested prompts from Appendix B or any other points that they feel are relevant to the case and discussion the group is having. The facilitator(s) should not feel required to discuss all the learning objectives, but rather focus on those that are relevant to the patient case and the questions the group may have. The learners should be encouraged to ask questions and guide the discussion in a way that is useful for their learning about feeding/nutrition in CMC.

This should take about 10 minutes.

At the bedside:

If time allows, and if the patient/family agrees, the group should go to the bedside to continue the discussion. The facilitator may point out parts of the feeding tube, the pump, extension tubing, etc. If the patient and/or family wants, they can participate in the discussion. The group can ask them questions about their experience with a feeding tube. Some possible questions can be found in Appendix B. This should take 5-10 minutes.

To conclude:

The facilitator may ask every member of the group to go around and list 1-2 take away points that they will remember as a result of the session. Learners should ask any final questions they may have. This should take 3-5 minutes.
