# Supplementary material for: Caring for Children With Medical Complexity: A Clinical, Patient-Focused Curriculum
Source: MedEdPORTAL. 2024 Jan 30;20:11380. doi: 10.15766/mep_2374-8265.11380 (PMC10825041; doi:10.15766/mep_2374-8265.11380)
Supplement: Supplementary file 1 — General Facilitator Guide.docxFeeding Nutrition Facilitator Objectives and Prompts.docxPain Irritability Facilitator Objectives and Prompts.docxFeeding Nutrition Case Example.docxPain Irritability Case Example.docxFeeding Nutrition Handout.docxPain Irritability Handout.docxFeeding Nutrition Evaluation.docxPain Irritability Evaluation.docx [file mep_2374-8265.11380-s001.zip › E. Pain Irritability Case Example.docx]

An Example Case of How to Discuss Pain/Irritability in CMC

The following is meant to serve as an example of how a learning session may run. We have provided an example patient case, example questions/prompts the facilitator may use to guide the session, and potential responses/discussion points that the facilitator would hope to elicit from the group. The following is to serve as a model of how a session might run, knowing that there are numerous ways the facilitator could use the patient case to discuss the following learning objectives.

**Learning Objectives*:**

By the end of this activity, learners will be able to:

1. Outline components of the history and physical examination that aid in evaluating pain in a child with medical complexity (CMC).
2. List different pharmacologic and nonpharmacologic interventions for pain in CMC.
3. Outline components of the history and physical examination that aid in recognizing and evaluating irritability in CMC.
4. List different pharmacologic and nonpharmacologic interventions for irritability in CMC.

** Depending on time and the course of the discussion, facilitators may choose to focus on some of the objectives, rather than covering all four*

The Patient Case:

After group introductions (2 minutes), the facilitator should ask one of the trainees to present the patient to the group, focusing on their past medical history, reason for admission, and hospital course thus far (approximately 5 minutes).

*Trainee: Our patient is a 13-year-old boy with history of prematurity (born at 25wk), neonatal stroke, epilepsy, spastic quadriplegia cerebral palsy, developmental delay, and oropharyngeal dysphagia with G-J tube dependence, initially presented with 3 days of fever and “irritability”. Parents noticed he was more uncomfortable with diaper changes. At baseline, he is non-verbal and dependent on his parents and nursing staff to take care of his basic activities. He is not continent of urine or stool. His seizures have generally been well controlled on his anti-epileptic regimen, with his last seizure being two years ago. He is admitted for pain management and further diagnostic work-up.*

Facilitated Discussion:

The facilitator will now lead an interactive discussion to try and discuss the learning objectives as they relate to the patient case. The following is an example of how the facilitator may guide the discussion, with the respective learning objectives in parentheses. This should take approximately 10-15 minutes.

**Facilitator: The parents reported the patient had 3 days of “irritability” prior to coming to the hospital. How might you investigate what this means? (Learning Objective #3)**

*Trainee: When taking the history from the parent/caregiver, you may clarify what irritability means to them. You might ask them questions like: How is the patient’s current behavior different from how they are at baseline? Since our patient is non-verbal, how do you know when he is uncomfortable, happy, tired, hungry, etc.?*

**Facilitator: Yes, using the parent/caregiver to further clarify the patient’s baseline mental status and how the patient is able to communicate what they are feeling is important. Besides clarifying the history with the parents/caregivers, are there other means of investigating why the patient’s behavior has changed? What about our patient’s history could be a clue to the etiology?**

*Trainee: You may look at the vital signs. If possible, you may try to see if there is a recent heart rate, blood pressure, and respiratory rate from when the patient was well, and compare it to now. You can ask the parent/caregiver to help with the physical exam by letting the team know if the patient seems more rigid or tense, or if they are less interactive than baseline. You should also do a complete physical exam from head to toe, especially if the patient is not able to localize any source/reason for the change in behavior. Our patient had a fever and was uncomfortable with movement, which could be a clue for an infectious etiology, localized to the lower extremities.*

**Facilitator: Now that we have discussed how we might use the history and physical exam to get at our diagnosis, what are some etiologies we might consider for our patient’s presentation?**

*Trainee: Although our patient has significant neurologic impairment, it would be important to consider common causes of discomfort like constipation or otitis media. Because of his cerebral palsy and developmental delay, he requires assistance to move, which could put him at risk for decubitus ulcers or a fracture, which could be painful. He has a fever and is G-J tube fed, but there could be a risk for aspiration pneumonia. He also has a fever and pain with diaper changes which could be a bone, joint, or soft tissue infection.*

**Facilitator: When considering the differential diagnosis of pain/irritability in a child with medical complexity, it is important to not only consider etiologies specific to their chronic condition, but also other common pediatric diagnoses. Let’s imagine you are called to the bedside overnight because the nurse is concerned that he seems uncomfortable. You still do not have a diagnosis yet for his change in behavior. There is nobody at the bedside to confirm whether he is uncomfortable. What would you do?**

*Trainee: I know there are different pain scales that you can use to quantify a patient’s pain, but I am not sure what they are or how to use them*.

**Facilitator**: **There are many different pain scales that can be used to quantify a patient’s pain. Today we will practice using the Revised FLACC which is in your handout (Appendix G). Let us review together the different components of this scale.**

Hands-on Demonstration:

At this point in the discussion, the facilitator or any other content experts present may want to use the information in the handout (Appendix G) to demonstrate how to use different pain scales. The facilitator may bring other materials that they feel appropriate for the session, and demonstrate them at this time.

Depending on time, the facilitator(s) could use additional suggested prompts from Appendix C or any other points that they feel are relevant to the case and discussion the group is having. The facilitator(s) should not feel required to discuss all the learning objectives, but rather focus on those that are relevant to the patient case and the questions the group may have. The learners should be encouraged to ask questions and guide the discussion in a way that is useful for their learning about pain/irritability in CMC.

This should take about 10 minutes.

At the bedside:

If time allows, and if the patient/family agrees, the group should go to the bedside to continue the discussion. The facilitator may point out parts of the patient’s physical exam, such as tone, mental status, strength, spasticity, etc. If the family/caregiver wants, they can participate in the discussion. The group can practice asking how they recognize when the patient is in pain or uncomfortable, what strategies they have utilized in the past when the patient’s behavior is not at baseline, etc. Refer to Appendix C for other suggested questions. This should take 5-10 minutes.

To conclude:

The facilitator may ask every member of the group to go around and list 1-2 take away points that they will remember as a result of the session. Learners should ask any final questions they may have. This should take 3-5 minutes.
