# Supplementary material for: Caring for Children With Medical Complexity: A Clinical, Patient-Focused Curriculum
Source: MedEdPORTAL. 2024 Jan 30;20:11380. doi: 10.15766/mep_2374-8265.11380 (PMC10825041; doi:10.15766/mep_2374-8265.11380)
Supplement: Supplementary file 1 — General Facilitator Guide.docxFeeding Nutrition Facilitator Objectives and Prompts.docxPain Irritability Facilitator Objectives and Prompts.docxFeeding Nutrition Case Example.docxPain Irritability Case Example.docxFeeding Nutrition Handout.docxPain Irritability Handout.docxFeeding Nutrition Evaluation.docxPain Irritability Evaluation.docx [file mep_2374-8265.11380-s001.zip › F. Feeding Nutrition Handout.docx]

Children with Medical Complexity Curriculum: Feeding/Nutrition

*This handout is intended to be distributed to learners during the session to be used during the hands-on portion of the discussion.*

**How to calculate formula and free water needs:**

|  | Age | Energy (kcal/kg/day) |
| --- | --- | --- |
| Infants | 2-3 mo | 102 |
|  | 4-6 mo | 82 |
|  | 7-12 mo | 89 |
|  | 13-35 mo | 82 |
| Boys | 3 yo | 85 |
|  | 4-5 yo | 70 |
|  | 6-7 yo | 64 |
|  | 8 yo | 59 |
|  | 9-11 yo | 49 |
|  | 12-13 yo | 44 |
|  | 14-16 | 39 |
|  | 17-18 | 37 |
|  | >18 yo | 36 |
| Girls | 3 yo | 82 |
|  | 4-5 yo | 65 |
|  | 6-7 yo | 61 |
|  | 8 yo | 59 |
|  | 9-11 yo | 42 |
|  | 12-13 yo | 40 |
|  | 14-16 | 33 |
|  | 17-18 | 31 |
|  | >18 yo | 34 |

1. Calculate the daily caloric needs using the Dietary Reference Intakes (DRI)^21^ values in the table
   1. Multiple the patient’s weight x the kcal/kg/day to get the calories needed in 24 hours
   2. Divide the total calories by the calorie concentration of the formula. This is the total volume of formula needed in 24 hours
2. Use the Holliday Segar Method^22^ to determine total maintenance fluids needs:

| Weight | Fluid per day |
| --- | --- |
| 1-10kg | 100mL/kg |
| 11-20kg | 1000mL + 50mL/kg for every kg >10kg |
| >20 kg | 1500mL + 20mL/kg for every kg >20kg |

1. To calculate free water needs
   1. Calculate amount of free water the patient is getting from their formula by multiplying the % free water of the formula x the total volume of formula
   2. Take the total fluids needs as determined by Holliday Segar method and subtract the free water from formula to determine the additional daily water needs

Example: 4-year-old, 15kg female, receiving G tube feeds with formula of 1.5cal/mL concentration, that is 77% free water

1. Daily calories: 65kcal/kg x 15= 975 kcal/day
   1. 975 calories/day / 1.5 calories/mL= 650mL/day
2. Fluid needs: 1000 mL + (50mL x 5) = 1250 mL/day
3. 0.77 x 650 mL = 500 mL of water in the formula patient is receiving
   1. 1250mL – 500 mL in formula= 750mL additional free water needed daily

**When to Initiate Tube Feeding^20^**

Generally used when there is inadequate or unsafe oral intake, and a functioning GI tract. Examples:

- Insufficient oral intake- anorexia, food aversion
- Malabsorption- cystic fibrosis, short bowl syndrome, pancreatic insufficiency
- Increased caloric needs- congenital heart disease, bronchopulmonary dysplasia
- Metabolic disease, intolerance to fasting
- Oral motor dysfunction- prematurity, neuromuscular or neurologic disease
- Abnormal gastrointestinal tract- congenital malformations, esophageal stenosis
- Injury/illness- burn, trauma, surgery

**Pre-pyloric versus Post-pyloric**

- Pre-pyloric feeding:
  - More physiologic
  - Patients can typically tolerate larger volumes and higher osmotic loads
  - Can compress feeds which allows for a more flexible feeding schedule
  - Has potential risk of pneumonia from aspiration of formula from the stomach
  - Caregivers can be taught how to replace G tube at home
- Post-pyloric feeding:
  - Can be useful when there is risk for aspiration pneumonia, intolerance to gastric feeding, severe gastroesophageal reflux, or recurrent emesis
  - May be especially helpful in children who are unable to protect their airways
  - Patients typically cannot tolerate the large volume or high osmotic loads of bolus feeding
  - Must be continuous feeding which means more time connected to a feeding pump
  - Must be replaced by specialist who placed the tube

**Types of Feeding Tubes**

1.
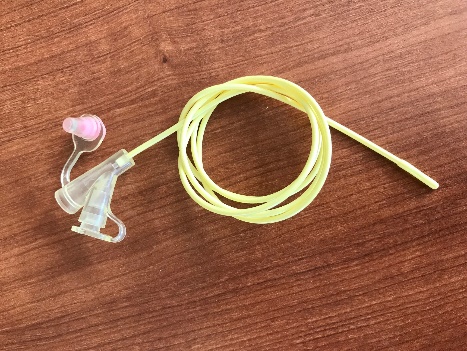
Nasogastric (NG)
   -
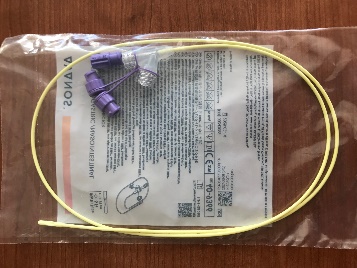
Short-term
   - Pre-pyloric
   - Used for feeding, medications
   - May be replaced at home by caregiver


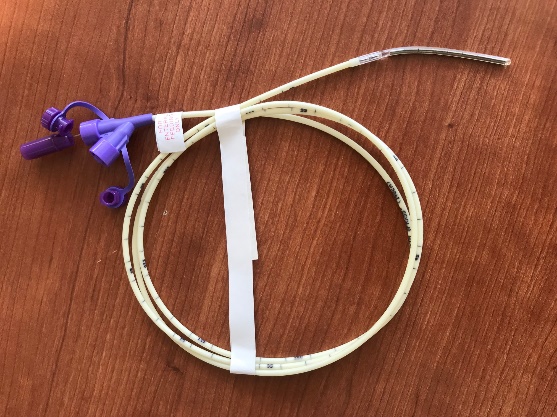


1. Nasoduodenal (ND)
   - Short-term
   - Post-pyloric
   - No access to stomach
   - Typically has a weighted distal tip
   - Typically utilize x-ray to confirm placement


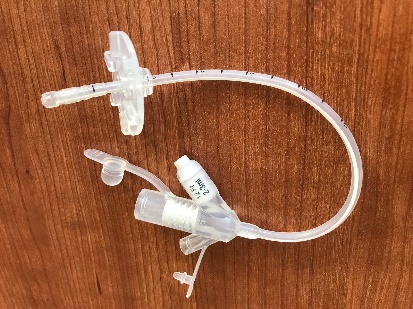


1. Gastrostomy (GT, G tube)
   - Long-term
   - Pre-pyloric
   - Placed surgically or Percutaneous Endoscopic Gastrostomy (PEG)
   -
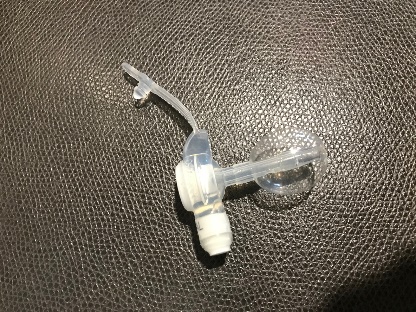
Long tubes (*above*) or Low-profile (button) tubes (*below*)
2.
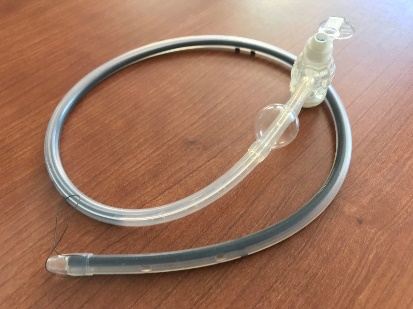
Gastrojejunostomy (GJT, G-J tube)
   - Long-term
   - Can access both stomach and small intestine through separate ports (*below*)
   - Single point of entry into abdomen
   - Limits reflux and aspiration when items delivered into jejunum
   -
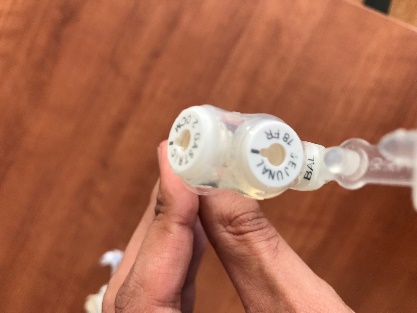
Feeds through the intestine are post-pyloric and are continuous with rare exceptions. Cannot tolerate bolus feeds
   - Gastric port allows for venting and medication administration
3. Jejunostomy (JT, J tube)
   - Long-term
   - No access to stomach
   - Feeds are post-pyloric and need to be continuous. Cannot tolerate bolus feeds
   - Surgically placed and not at risk of coiling in intestines
   - Provides direct access to jejunum

References:

1. Singhal S, Baker SS, Bojczuk GA, Baker RD. Tube feeding in children. *Pediatr Rev*. 2017;38(1):23-34. <https://doi.org/10.1542/pir.2016-0096>
2. Otten JJ, Hellwig JP, Meyers LD, eds. *DRI, Dietary Reference Intakes: The Essential Guide to Nutrient Requirements*. National Academies Press; 2006.
3. Holliday MA, Segar WE. The maintenance need for water in parenteral fluid therapy. *Pediatrics*. 1957;19(5):823-832.
