# Supplementary material for: Caring for Children With Medical Complexity: A Clinical, Patient-Focused Curriculum
Source: MedEdPORTAL. 2024 Jan 30;20:11380. doi: 10.15766/mep_2374-8265.11380 (PMC10825041; doi:10.15766/mep_2374-8265.11380)
Supplement: Supplementary file 1 — General Facilitator Guide.docxFeeding Nutrition Facilitator Objectives and Prompts.docxPain Irritability Facilitator Objectives and Prompts.docxFeeding Nutrition Case Example.docxPain Irritability Case Example.docxFeeding Nutrition Handout.docxPain Irritability Handout.docxFeeding Nutrition Evaluation.docxPain Irritability Evaluation.docx [file mep_2374-8265.11380-s001.zip › G. Pain Irritability Handout.docx]

Children with Medical Complexity Curriculum: Pain/Irritability

*This handout is intended to be distributed to learners during the session to be used during the hands-on portion of the discussion.*

**Distinction**

- **Children with Medical Complexity (CMC)**: a heterogeneous patient population defined by having 1) chronic conditions, 2) functional limitations often associated with dependence on technology, 3) high health care utilization, and 4) substantial health care service needs^1^
  - *Note this does not comment on their neurologic or intellectual function. CMC may or may not have neurologic impairment*
- **Children with Severe Neurologic Impairment (SNI)**: those with severe impairment of the central nervous system that results in lifelong intellectual disability and limited verbal communication, which often coexists with motor impairment^25^

**Definitions^23, 24^**

| Term | Definition |
| --- | --- |
| Pain | An unpleasant sensory and emotional experience associated with actual or potential tissue damage |
| Irritability | Abnormal responsiveness to stimuli; may be in response to pain, fright, drug, an emotional situation, or a medical condition |
| Neuroirritability | Children with SNI who exhibit persistent or recurrent pain behaviors after either non-yielding assessment or failed management of potential nociceptive sources. This suggests the CNS may be a source of persistent pain features |
| Nociceptive pain | Pain that arises from actual/potential damage to non-neural tissue and is due to activation of nociceptors |
| Neuropathic pain | Pain caused by a lesion or disease of the somatosensory nervous system |
| Allodynia | Pain due to a stimulus that does not usually provoke pain |
| Hyperalgesia | Increased pain from a stimulus that usually provokes pain |
| Dystonia | Involuntary sustained or intermittent muscle contractions cause twisting and repetitive movements, abnormal postures, or both. Pain can trigger and worsen dystonia |
| Spasticity | Velocity-dependent increase in muscle tone that results in muscles that are resistant to movement |
| Muscle spasms | Sudden involuntary contraction of a muscle or group of muscles. Can lead to arching, stiffening, and clonus |

**Pain Scales to use in children with SNI**

**Revised FLACC (Face, Legs, Activity, Cry, Consolability) Scale^26^**

- Each category is scored from 0-2, total score between 0 and 10
- Validated as a measure of pain in children with varying degrees of neurologic impairment

| Categories | 0 | 1 | 2 |
| --- | --- | --- | --- |
| Face | No particular expression or smile | Occasional grimace/frown; withdrawn or disinterested; appears sad or worried | Consistent grimace or frown; frequent/constant quivering chin, clenched jaw; distressed-looking face; expression of fright or panic |
| Legs | Normal position or relaxed; usual tone and motion to limbs | Uneasy, restless, tense; occasional tremors | Kicking, or legs drawn up; marked increase in spasticity, constant tremors or jerking |
| Activity | Lying quietly, normal position, moves easily; regular, rhythmic respirations | Squirming, shifting back and forth, tense or guarded movements; mildly agitated (e.g. head back and forth, aggression); shallow, splinting respirations, intermittent sighs | Arched, rigid or jerking; severe agitation; head banging; shivering (not rigors), breath holding, gasping or sharp intake of breaths, severe splinting |
| Cry | No cry/verbalization | Moans or whimpers; occasional complaint; occasional verbal outburst or grunt | Crying steadily, screams or sobs, frequent complaints; repeated outbursts, constant grunting |
| Consolability | Content and relaxed | Reassured by occasional touching, hugging, or being talked to. Distractable | Difficult to console or comfort; pushing away caregiver, resisting care or comfort measures |

**Individualized Numeric Rating Scale (INRS)^27^**

- Personalized pain-assessment tool based on parent’s knowledge of the child
- Parents and caregivers identify behaviors/signs that correlate to no pain to the worst pain on a scale of 0 to 10

0 1 2 3 4 5 6 7 8 9 10

**Interventions for Pain^23, 25^**

- Non-pharmacologic interventions
  - Swaddling, cuddling, rocking, massage
  - Complementary approach for anxiety management: hypnotherapy, relaxation therapy, cognitive behavioral therapies,
  - Use of supportive equipment: supportive pillows, cushions, etc.
  - Physical, occupational and speech/swallow therapies
  - Warm or cold packs, massage, acupressure/acupuncture, essential oils and aromatherapy, Reiki
  - Music, art, animal therapy, child life
  - Vibratory stimulation: vibrating mats or pillows, chest-wall oscillation vest therapy
  - Gastrostomy tube venting
  - Braces and positioning, passive stretching, warm baths (especially for spasms)
- Pharmacologic interventions
  - Acetaminophen
- Used for mild pain
- Has central acting analgesic and antipyretic properties
- Crosses blood brain barrier easily
- Risks include hepatotoxicity
  - Non-Steroidal Anti-Inflammatory Drugs (NSAIDs)
- Used for mild and moderate pain and inflammation
- Strong opioid sparing properties
- Cyclooxygenase and prostaglandin inhibition
- Risks for renal, gastrointestinal toxicity, and bleeding
  - Opioids
    - Used in acute nociceptive pain, acute breakthrough pain despite use of standing medications for chronic pain, sedation weans, and autonomic storms
    - Morphine sulfate and Hydromorphone (short acting), Fentanyl
    - Risks include respiratory depression, nausea, vomiting, constipation, sedation, pruritus, pharmacologic tolerance, opioid induced hyperalgesia
  - Gabapentinoids
    - Gabapentin and pregabalin
    - Used for neuropathic pain and hyperalgesia
    - Often used as first-line medication in recurrent pain given general safety
    - Risks include sedation, respiratory depression (especially if used with opioids, antihistaminics etc.), serotonin syndrome, neuropsychiatric changes
  - NMDA Antagonists
    - Ketamine and Methadone
    - Ketamine can also be used for procedural sedation
    - Methadone is a long-acting opioid
    - Risks include
      - Ketamine: tachycardia, bronchospasm
      - Methadone: QT prolongation, similar risks to opioids
  - Tricyclic Antidepressants (TCAs)
    - Nortriptyline and Amitriptyline
    - Used for neuropathic pain and hyperalgesia. Often used as second-line medication after gabapentinoids for recurrent pain behaviors
    - Risks include anticholinergic properties- may have beneficial effect of decreased secretion, serotonin syndrome, cardiac dysrhythmia
  - Clonidine
    - Used in spasticity and autonomic dysfunction, potentially mild analgesia

Risks include hypotension (avoid abruptly discontinuing as rebound hypertension may follow), sedation (may be beneficial in those with disrupted sleep)

- - Anticonvulsants
    - Valproic acid, carbamazepine, oxcarbazepine, lamotrigine, topiramate
    - Used in adults with neuropathic pain as third- or fourth-line
    - Role in children is unclear as most studies are performed in adult populations
  - Benzodiazepines
    - Clonazepam, Lorazepam, Midazolam
    - Used for spasticity, dystonia, seizures, dysautonomia, agitation, sleep
    - Avoid sudden cessation to prevent withdrawal
    - Risks include jitteriness, agitation, increased heart rate, muscle cramps, GI upset, tolerance
  - Antipsychotics
    - Haloperidol, Risperidone
    - Used for agitation, delirium, self-injurious behaviors
    - Risks include extrapyramidal symptoms
- Spasticity Management
  - Baclofen
    - GABA agonist
    - Can be provided as intrathecal pump
    - Also approved for management of dystonia
  - Tizanidine
  - Clonidine
  - Dantrolene
  - Benzodiazepines for intermittent or short-term use
  - Botulinum toxin injections

References:

1. Hauer J, Houtrow AJ; Section on Hospice and Palliative Medicine Council on Children With Disabilities. Pain assessment and treatment in children with significant impairment of the central nervous system. *Pediatrics*. 2017;139(6):e20171002. <https://doi.org/10.1542/peds.2017-1002>
2. Terminology. International Association for the Study of Pain. Accessed October 19, 2022. <https://www.iasp-pain.org/resources/terminology/>
3. Giordano M, Knipper E, Melwani A. Clinical progress note: perioperative pain control in hospitalized pediatric patients. *J Hosp Med*. 2021;16(6):358-360. <https://doi.org/10.12788/jhm.3388>
4. Malviya S, Voepel-Lewis T, Burke C, Merkel S, Tait AR. The revised FLACC observational pain tool: improved reliability and validity for pain assessment in children with cognitive impairment. *Pediatr Anesth*. 2006;16(3):258-265. <https://doi.org/10.1111/j.1460-9592.2005.01773.x>
5. Solodiuk JC, Scott-Sutherland J, Meyers M, et al. Validation of the Individualized Numeric Rating Scale (INRS): a pain assessment tool for nonverbal children with intellectual disability. *Pain*. 2010;150(2):231-236. <https://doi.org/10.1016/j.pain.2010.03.016>
