# Supplementary material for: Caring for Children With Medical Complexity: A Clinical, Patient-Focused Curriculum
Source: MedEdPORTAL. 2024 Jan 30;20:11380. doi: 10.15766/mep_2374-8265.11380 (PMC10825041; doi:10.15766/mep_2374-8265.11380)
Supplement: Supplementary file 1 — General Facilitator Guide.docxFeeding Nutrition Facilitator Objectives and Prompts.docxPain Irritability Facilitator Objectives and Prompts.docxFeeding Nutrition Case Example.docxPain Irritability Case Example.docxFeeding Nutrition Handout.docxPain Irritability Handout.docxFeeding Nutrition Evaluation.docxPain Irritability Evaluation.docx [file mep_2374-8265.11380-s001.zip › I. Pain Irritability Evaluation.docx]

**Children with Medical Complexity (CMC) Curriculum Evaluation**

Training Level (please circle one): Student PGY1 PGY2 PGY3

Have you been on Team 1 before? Yes No

Have you participated in a Children with Medical Complexity session before? Yes No

**Curriculum Evaluation**

|  | Strongly Agree | Agree | Disagree | Strongly Disagree |
| --- | --- | --- | --- | --- |
| A patient-centered curriculum is an engaging way to learn. |  |  |  |  |
| A patient-centered curriculum is an effective way to learn. |  |  |  |  |
| This session was relevant to my clinical practice. |  |  |  |  |
| The amount of information was appropriate. |  |  |  |  |
| I would like to have more sessions like this in the future. |  |  |  |  |

**Self-Evaluation**

| **By participating in this learning experience…** | Strongly Agree | Agree | Disagree | Strongly Disagree |
| --- | --- | --- | --- | --- |
| my knowledge about topics pertinent to CMC has increased. |  |  |  |  |
| my ability to effectively care for CMC has improved. |  |  |  |  |
| my confidence in caring for CMC has improved. |  |  |  |  |
| I will provide better care to my patients. |  |  |  |  |

Please describe at least two ways in which your practice may change after participating in this session.

Mark how you would have answered the following **PRIOR** to today’s session.

|  | Beginner | Some knowledge | Competent | Highly skilled | Expert |
| --- | --- | --- | --- | --- | --- |
| My ability to evaluate pain in a child with medical complexity (CMC). |  |  |  |  |  |
| My understanding of the different interventions for pain for a CMC. |  |  |  |  |  |
| My ability to recognize and evaluate irritability in a child with medical complexity. |  |  |  |  |  |
| My understanding of the different interventions for irritability in a CMC. |  |  |  |  |  |

Now **AFTER** participating in today’s session, please answer the following.

|  | Beginner | Some knowledge | Competent | Highly skilled | Expert |
| --- | --- | --- | --- | --- | --- |
| My ability to evaluate pain in a child with medical complexity. |  |  |  |  |  |
| My understanding of the different interventions for pain for a CMC. |  |  |  |  |  |
| My ability to recognize and evaluate irritability in a child with medical complexity. |  |  |  |  |  |
| My understanding of the different interventions for irritability in a CMC. |  |  |  |  |  |

Please list suggestions for how we can improve this learning experience in the future.
